# Algae-Produced Pfs25 Elicits Antibodies That Inhibit Malaria Transmission

**DOI:** 10.1371/journal.pone.0037179

**Published:** 2012-05-16

**Authors:** James A. Gregory, Fengwu Li, Lauren M. Tomosada, Chesa J. Cox, Aaron B. Topol, Joseph M. Vinetz, Stephen Mayfield

**Affiliations:** 1 Division of Biological Sciences, and the San Diego Center for Algae Biotechnology, University of California San Diego, California, United States of America; 2 Division of Infectious Disease, Department of Medicine, University of California San Diego, La Jolla, California, United States of America; University of Copenhagen, Denmark

## Abstract

Subunit vaccines are significantly more expensive to produce than traditional vaccines because they are based primarily on recombinant proteins that must be purified from the expression system. Despite the increased cost, subunit vaccines are being developed because they are safe, effective, and can elicit antibodies that confer protection against diseases that are not currently vaccine-preventable. Algae are an attractive platform for producing subunit vaccines because they are relatively inexpensive to grow, genetically tractable, easily scaled to large volumes, have a short generation time, and are devoid of inflammatory, viral, or prion contaminants often present in other systems. We tested whether algal chloroplasts can produce malaria transmission blocking vaccine candidates, *Plasmodium falciparum* surface protein 25 (Pfs25) and 28 (Pfs28). Antibodies that recognize Pfs25 and Pfs28 disrupt the sexual development of parasites within the mosquito midgut, thus preventing transmission of malaria from one human host to the next. These proteins have been difficult to produce in traditional recombinant systems because they contain tandem repeats of structurally complex epidermal growth factor-like domains, which cannot be produced in bacterial systems, and because they are not glycosylated, so they must be modified for production in eukaryotic systems. Production in algal chloroplasts avoids these issues because chloroplasts can fold complex eukaryotic proteins and do not glycosylate proteins. Here we demonstrate that algae are the first recombinant system to successfully produce an unmodified and aglycosylated version of Pfs25 or Pfs28. These antigens are structurally similar to the native proteins and antibodies raised to these recombinant proteins recognize Pfs25 and Pfs28 from *P. falciparum*. Furthermore, antibodies to algae-produced Pfs25 bind the surface of *in-vitro* cultured *P. falciparum* sexual stage parasites and exhibit transmission blocking activity. Thus, algae are promising organisms for producing cysteine-disulfide-containing malaria transmission blocking vaccine candidate proteins.

## Introduction

Vaccines have transformed medicine and dramatically improved human health. However, traditional vaccines that consist of attenuated or killed pathogens are not always practical or effective. A more modern strategy is to present specific immunogenic subunits from a pathogen to the immune system [1], [2]. Antibodies raised to these subunit vaccines, which are generally recombinant proteins or synthesized peptides, recognize native proteins from the appropriate pathogen and confer protection to the vaccinated individual. This reductionist approach is generally safer than traditional vaccines because there is no risk of a recombinant protein becoming virulent, and protein subunits promote a more directed immune response because of decreased antigenic competition. In the United States, vaccines cost $10–120 per dose (Center for Disease Control; [3]) with subunit vaccines being the most expensive. Though impoverished nations would benefit the most from potential subunit vaccines for malaria, dengue, and other diseases for which traditional vaccines do not exist, the cost of recombinant subunit vaccines is too expensive for use in these areas [4]. Thus, cheaper methods for producing subunit vaccines should be a priority if we are to reduce the heavy disease burdens found in low income countries.

Every system used to produce recombinant proteins has its own advantages and disadvantages with respect to cost, protein folding, yield, ease of manipulation, and scalability, which must be carefully weighed against the specific application and structure of the antigen. One strategy for reducing the cost of subunit vaccines is to use plants as the expression system [5], [6]. Photosynthetic growth, combined with high recombinant protein yields and economy of scale, result in a relatively inexpensive production platform [7]. In plants, both the nuclear and plastid genome can be transformed with genes that encode subunit vaccines. Indeed, several plant-made pharmaceuticals are currently in clinical trials [8]. Microalgae, which are essentially single-celled water-borne plants, are easily manipulated, have much shorter generation times than terrestrial plants, and can be cultured in complete containment at a relatively low cost. The development of algal biofuels has also spurred interest in potential co-products that could be produced concurrently, thus driving down the cost of both the fuel and any associated algae-produced protein. The chloroplast of the eukaryotic green microalgae, *Chlamydomonas reinhardtii*, is genetically tractable and has been used to produce human monoclonal antibodies [9], [10], human protein therapeutics [11], and subunit vaccines [12], [13]. However, the diversity of recombinant proteins that can be produced in algal chloroplasts is still not well-explored. We tested whether algal chloroplasts can produce malaria transmission blocking vaccines (TBV) by producing the well-studied structurally complex malaria TBV candidates, *Plasmodium falciparum* surface protein 25 (Pfs25) and 28 (Pfs28).

The *Plasmodium* parasite that is the causative agent of malaria can only be transmitted via the female *Anopheles* mosquito. There it undergoes the sexual stages of development that eventually lead to the infectious sporozoites that reside in the mosquito salivary gland [14]. Several conserved proteins involved in this process, in particular Pfs25, are of great interest because antibodies raised to these proteins block parasite maturation leading to sporozoites, thus preventing subsequent transmission to a human host [15]. A TBV would complement current vector control strategies and increase the efficacy of the protective RTS/S vaccine [16], [17], but a safe and effective TBV has not been realized despite its initial conception in 1976 [18], [19].

Pfs25 and Pfs28 are structurally complex aglycosylated outer membrane proteins that contain four tandem epidermal growth factor-like (EGF) domains, each with several disulfide bonds. The biological limitations of traditional recombinant systems have made it difficult to reproduce the complex structures of these proteins. Attempts to produce conformationally correct Pfs25 in *Escherichia coli* failed [20], and yeast-produced Pfs25 has multiple conformations [21], [22] and caused an allergic reaction during human clinical trials [23]. More recently, Pfs25 was produced in a tobacco transient expression system and is a promising TBV candidate [24], but like yeast, requires mutations in the nucleotide sequence to prevent post-translational glycosylation. Furthermore, transient expression mediated by *Agrobacterium* infiltration may not be as suitable for large scale production as algae. Like yeast and tobacco, the chloroplast of green algae can fold complex proteins and make disulfide bonds, but lacks the machinery for glycosylation. Here we describe the physical and immunological characteristics of algae-produced Pfs25 and Pfs28.

## Results

### Production and purification of recombinant Pfs25 and Pfs28 in algal chloroplasts

**Figure 1:**
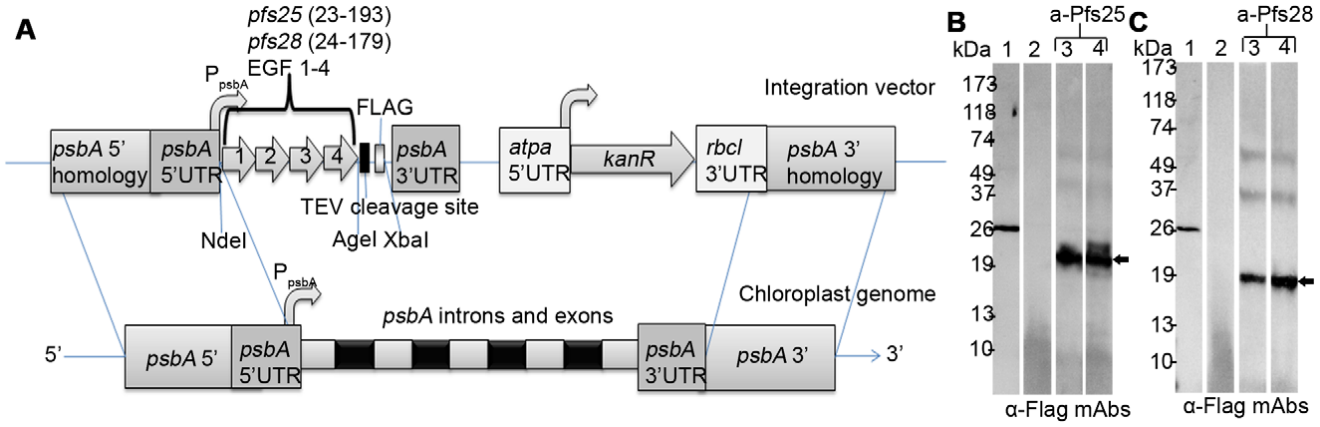
Diagram of chloroplast transformation vector and Western blots of *C. reinhardti* transformed with vectors containing codon optimized *pfs25* or *pfs28*. (A) The codon optimized nucleotide sequences corresponding to EGF domains 1–4 of *pfs25* and *pfs28* were separately cloned into an open reading frame that is upstream of a TEV protease site and FLAG epitope. Transgenes were integrated at the *psbA* locus by homologous recombination. Gene expression is driven by the *psbA* promoter and mRNA is stabilized by the *psbA* 5′ and 3′ untranslated regions (UTRs). (B) Western blot analysis of purified algae-produced HMGB1 containing a FLAG epitope (lane 1), lysate of untransformed parental strain (lane 2), lysate of *C. reinhardtii* containing *a-pfs25* total protein (lane 3) and soluble protein (lane 4) probed with anti-FLAG mAbs. (C) Western blot using anti-FLAG mAbs of purified algae-produced HMGB1 containing a FLAG epitope (lane 1), lysate of untransformed parental strain (lane 2), lysate of C. reinhardtii containing *a-pfs28* total protein (lane 3) and soluble protein (lane 4) probed with anti-FLAG mAbs.

We synthesized genes encoding *pfs25*(Ala_22_ – Thr_193_) and *pfs28*(Val_24_ – Pro_179_) with a codon bias that resembles the *C. reinhardtii* chloroplast codon usage (hereafter referred to as a-*pfs25* and a-*pfs28*). Codon optimization of heterologous genes for expression in the chloroplast was previously shown to increase protein yields [25]. Synthetic *a-pfs25* and *a-pfs28* each contain four EGF-like domains and a C-terminal FLAG tag for ease of detection and purification, but lack the native signal sequence and GPI-anchor sequence. The expected codon adaptation index (eCAI), which quantitates the codon bias of a transgene against a reference set, is 0.878 (p<.01) and 0.892 (p<. 01) for *a-pfs25* and *a-pfs25* respectively [26]. CAI values range from zero to one where a score of one indicates that every instance of an amino acid is encoded by the most common codon in the reference codon table [27]. *A*-*pfs25* and a-*pfs28* were synthesized and separately cloned into a chloroplast expression cassette that replaces endogenous *psbA* through homologous recombination such that transgene expression is controlled by the *psbA* promoter and 5′ and 3′ untranslated regions (UTRs; Fig. 1A). *C. reinhardtii* chloroplasts were transformed by particle bombardment and a-*pfs25* and a-*pfs28* were detected by PCR (data not shown). Transformed *C. reinhardtii* were screened for a-Pfs25 (Fig. 1B – arrow) and a-Pfs28 (Fig. 1C – arrow) protein by Western blot. A-Pfs25 and a-Pfs28 accumulate in chloroplasts at 0.5% and 0.2% total soluble protein, respectively, as determined by ELISA (see materials and methods).

**Figure 2:**
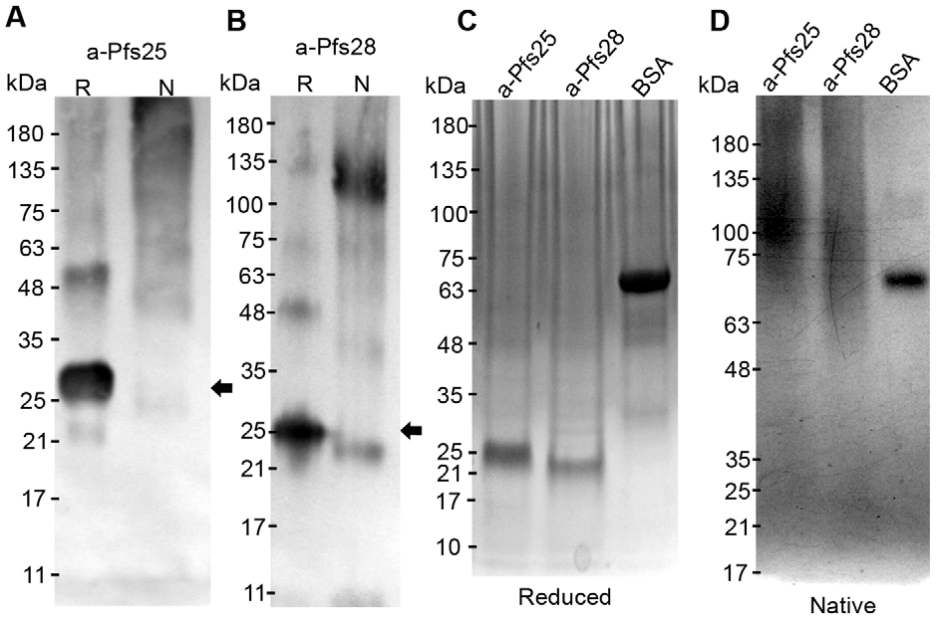
Immunoblot and Coomassie-blue stain of algae-produced Pfs25 and Pfs28 analyzed by SDS and Native-PAGE. Five micrograms of reduced and non-reduced affinity purified (A) a-Pfs25 and (B) a-Pfs28 were resolved by SDS-PAGE, transferred to nitrocellulose, and detected with anti-FLAG mAbs. (C) Five micrograms of reduced a-Pfs25, a-Pfs28, and BSA were resolved by SDS-PAGE and stained with Coomassie-blue. (D) Five micrograms of non-reduced a-Pfs25, a-Pfs28, and BSA were resolved by Native-PAGE and stained with Coomassie-blue. (R – reduced, N – non-reduced).

A-Pfs25 and a-Pfs28 were affinity purified using anti-FLAG M2 affinity resin (see materials and methods), positively identified by mass spectrometry (data not shown), and analyzed by Western blot (Fig. 2A–B). The predominant band in reduced a-Pfs25 and a-Pfs28 migrated near their predicted sizes of 21.4 kDa (Fig. 2A – arrow) and 20.2 kDa (Fig. 2B – arrow), respectively. The sizes of the larger bands suggest that a-Pfs25 and a-Pfs28 could be running as dimers. The monomeric form of a-Pfs25 and a-Pfs28 is diminished in unreduced samples and appears at larger, less well-defined molecular species. Similar to Western blot analysis, Coomassie-blue staining of reduced samples resolved by SDS-PAGE shows a single predominant band with few obvious impurities for both a-Pfs25 and a-Pfs28 (Fig. 2C). Similar results were obtained with silver staining (Fig. S1). A-Pfs25 and a-Pfs28 appear as larger less defined molecular weight complexes when analyzed by native-PAGE (Fig. 2D). The apparent larger size of a-Pfs25 and a-Pfs28 in the non-reduced and native gels could be the result of multimerization that form because both proteins are rich in β-strand secondary structures that are known to interact [28]. Indeed, multimerization of recombinant *Plasmodium* surface proteins was recently observed [29], [30]. Thus, algal chloroplasts produce a single protein that is the appropriate size for a-Pfs25 and a-Pfs28, and those monomers appear to assemble into higher molecular weight aggregates that could have multiple conformations.

### Structural characterization of a-Pfs25 and a-Pfs28

**Figure 3:**
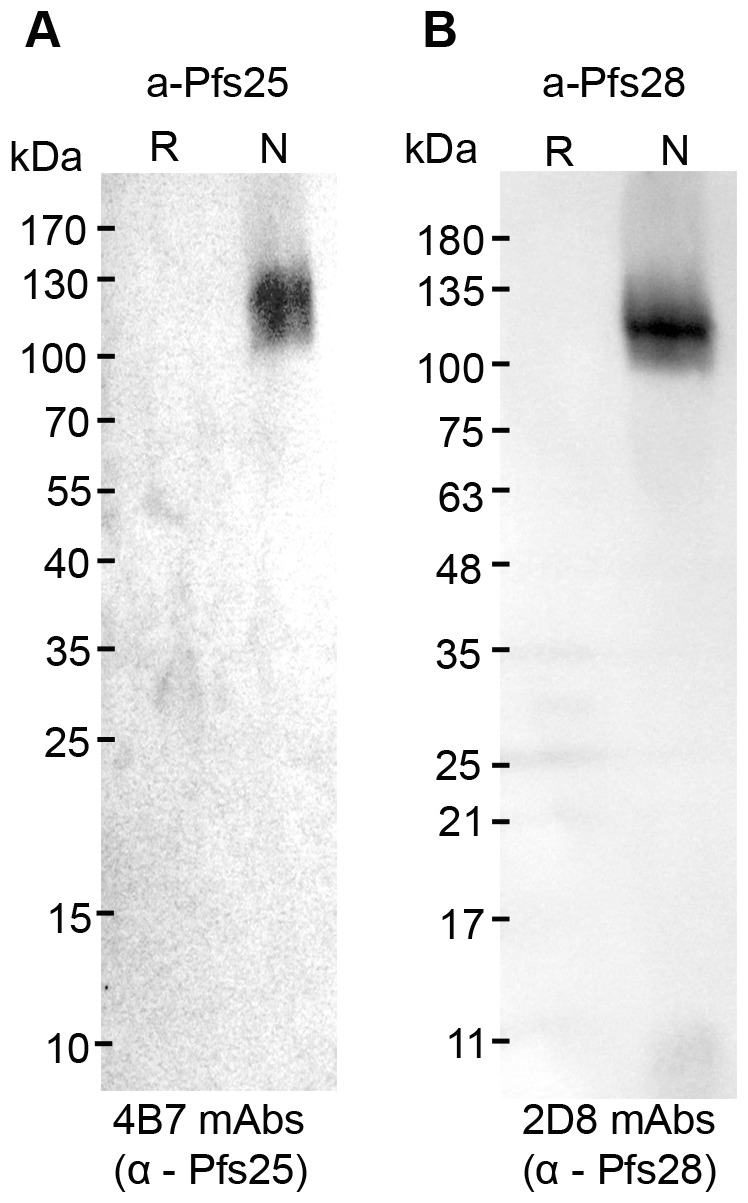
Immunoblot of reduced and non-reduced algae-produced Pfs25 and Pfs28 with monoclonal transmission blocking antibodies. Reduced and non-reduced a-Pfs25 and a-Pfs28 were resolved by SDS-PAGE, transferred to nitrocellulose, and detected with (A) anti-Pfs25 4B7 mAb and (B) anti-Pfs28-2D8 mAbs, respectively.

A-Pfs25 and a-Pfs28 were analyzed for the presence of epitopes found in native Pfs25 and Pfs28 by Western blot using transmission blocking monoclonal antibodies 4B7 (Pfs25, [31]) and 2D8 (Pfs28, a gift from David Narum, NIAID [32]), which were previously shown to recognize epitopes only present on properly folded Pfs25 and Pfs28, respectively(Fig. 3). Anti-Pfs25-4B7 mAbs recognize a β-hairpin epitope within the ILDTSNPVKT peptide sequence of the third EGF-like domain of native Pfs25 [33], [34]. Anti-Pfs25-4B7 mAbs binds a-Pfs25 as a band that migrates between 100 and 130 KDa in non-reduced samples (Fig. 3A). Unlike anti-FLAG mAbs (Fig. 2), anti-Pfs25-4B7 does not recognize reduced a-Pfs25, which is consistent with recognition being conformationally dependent. Similarly, anti-Pfs28-2D8 mAbs recognize a-Pfs28 in non-reduced, but not reduced samples, as a larger molecular weight species (Fig. 3B). Thus, a-Pfs25 and a-Pfs28 contain epitopes present on native Pfs25 and Pfs28, respectively.

**Figure 4:**
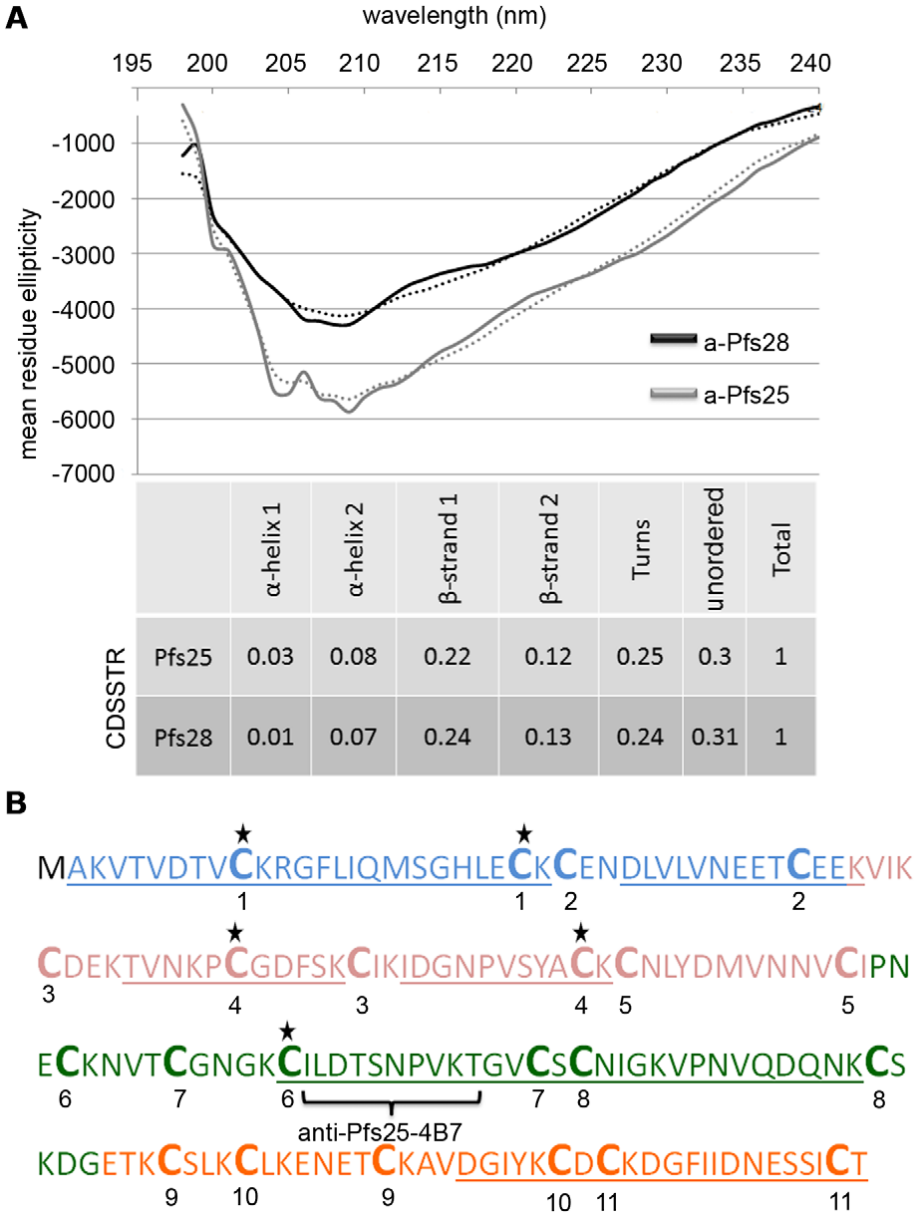
Structural analysis of algae-produced Pfs25 and Pfs28. (A) Far-UV circular dichroism spectra of algae-produced Pfs25 (Gray) and Pfs28 (Black). Spectra shown are measured mean residue ellipticity (solid lines) and best fit (dotted lines) using CDSSTR. The contribution of each secondary structure to the overall spectra as predicted by CDSSTR is shown as fractions. (B) Analysis of protected cysteines in a-Pfs25 by tandem mass spectrometry. The peptide sequence of Pfs25 from Ala_22_ to Thr_193_ is shown with EGF-like domain 1 (blue), 2(pink), 3(green), 4(orange). Peptides detected by mass spectrometry are underlined and protected cysteines are marked by stars. Predicted disulfide linkages between cysteines are marked numerically (i.e. the two cysteines labeled 1 form a dilsulfide linkage and so on). The epitope recognized by anti-Pfs25 4B7 mAb is indicated.

We assessed the secondary structure of purified a-Pfs25 and a-Pfs28 using circular dichroism (CD) spectroscopy(Fig. 4A). The crystal structure of yeast-produced *Plasmodium vivax* surface protein 25 (Pvs25), a homologue of Pfs25, has two central β-strands separated by a turn [35]. Bioinformatic analysis of Pfs25 and Pfs28 suggest their structures are similar [33]. Deconvolution of CD spectra with CDSSTR [36], [37], which estimates the contribution of each secondary structure to the overall spectrum, predicts that a-Pfs25 and a-Pfs28 are composed of nearly 60% β-strands and turns, 10% α-helix, and 30% unordered peptide. Thus, the CD spectra reveal that a-Pfs25 and a-Pfs28 have comparable secondary structures that are predicted to be primarily β-strands and turns, which is consistent with the crystal structure of Pvs25.

We analyzed free cysteine residues in a-Pfs25 by mass spectrometry to determine which residues are bridged by disulfide bonds (see materials and methods). Briefly, a-Pfs25 was treated with iodoacetamide to carboxymethylate free cysteines followed by trypsin digestion. Tryptic peptides were analyzed by high pressure liquid chromatography (HPLC) coupled to tandem mass spectrometry (LC-MS-MS) using nanospray ionization. Presumably, cysteines that remained unmodified after treatment with iodoacetamide were protected from modification because they were in disulfide bonds. The detected peptides covered approximately 64% of the Pfs25 sequence (Fig. 4B – underlined). A-Pfs25 digested with GluC and chymotrypsin did not add to the total coverage (data not shown). When compared to the disulfide bonds identified in crystallized yeast-produced Pvs25, we found that disulfide bonds 1, 4, and 6 are intact while 2, 7, 8, 10, and 11 may not be completely formed in a-Pfs25. Peptides for the remaining disulfide bonds were not detected. Hence, *C. reinhardtii* chloroplasts form disulfide bonds in a-Pfs25, but it may not be as extensively bridged by disulfide bonds as yeast-produced Pfs25.

### Antibodies from mice vaccinated with a-Pfs25 or a-Pfs28 recognize native parasite proteins

**Figure 5:**
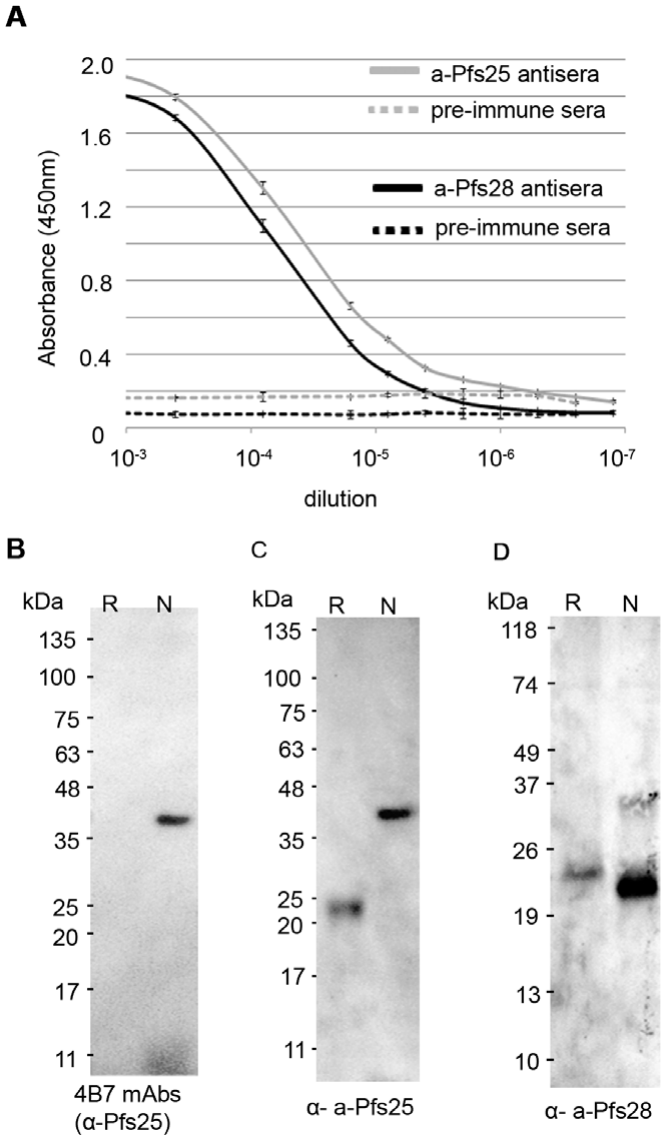
Analysis of antibodies from mice immunized with algae-produced Pfs25 or Pfs28. (A) ELISA titers of mouse anti-sera elicited by algae-produced Pfs25 and Pfs28. Mice were immunized with affinity purified a-Pfs25 or a-Pfs28 using complete Freund's adjuvant followed by boosters with incomplete Fruend's adjuvant by intraperitoneal injection. Pooled sera was serially diluted and tested in triplicate against the corresponding algae-produced Pfs antigen; error bars are one standard deviation. Prebleed sera were tested as a negative control; error bars are four standard deviations. (B–D) Western blot analysis of *P. falciparum* mixed sexual stage lysates with anti-Pfs25-4B7 mAbs, a-Pfs 25 antisera, or a-Pfs28 antisera. Reduced and non-reduced sexual stage lysates were resolved by SDS-PAGE and transferred to nitrocellulose. Blots were probed with (B) anti-Pfs25-4B7 mAbs, (C) antibodies raised to a- Pfs25, and (D) antibodies raised to a-Pfs28. (R – reduced, N – non-reduced).

Balb/c mice were immunized with affinity purified a-Pfs25 or a-Pfs28 using complete Freund's adjuvant for the first vaccination and incomplete Freund's adjuvant for subsequent vaccinations (see materials and methods). Antibody titers were measured by ELISA against affinity purified a-Pfs25 or a-Pfs28 (Fig. 5A). Sera from mice immunized with a-Pfs25 and a-Pfs28 contained high titers of antibodies for a-Pfs25 and a-Pfs28 respectively. Pre-immune sera showed no IgG response to either of these proteins.

We tested the specificity of antibodies raised to algae produced Pfs antigens to native Pfs25 or Pfs28 in sexual stage parasite lysates. Reduced and non-reduced *P. falciparum* sexual stage lysates were probed with anti-Pfs25-4B7 mAbs, anti-Pfs28-2D8 mAbs, and with serum from mice injected with a-Pfs25 or a-Pfs28. As expected, anti-Pfs25-4B7 recognized a band in the non-reduced sample, but not the reduced sample, which is consistent with anti-Pfs25-4B7 recognizing only conformationally-correct Pfs25 (Fig. 4B). Sera from mice injected with a-Pfs25 recognized a 25 kDa band in reduced *P. falciparum* lysates, which is consistent with molecular weight of native Pfs25, and an approximately 40 KDa band that is identical in size to the band recognized by anti-Pfs25-4B7 in non-reduced lysates (Fig. 5B & C). Antibodies raised to a-Pfs28 recognized an approximately 22 KDa band in both reduced and non-reduced parasite lysates (Fig. 5D), but required ten-fold more parasite lysate than a-Pfs25 antisera for detection. This could be due to lower Pfs28 protein abundance or due to lower affinity antibodies. We did not detect Pfs28 with anti-Pfs28-2D8 mAbs (data not shown) indicating Pfs28 is indeed less abundant than Pfs25 in our *in vitro* cultured parasites. Importantly, the size of the protein recognized by a-Pfs28 antisera is consistent with antibodies that were previously shown to recognize Pfs28 in sexual stage parasites [38]. Thus, antisera to both a-Pfs25 and a-Pfs28 recognize native parasite proteins of the appropriate size and a-Pfs25 antisera recognize a band that identical to the transmission blocking anti-Pfs25 4B7 mAbs in non-reduced samples.

**Figure 6:**
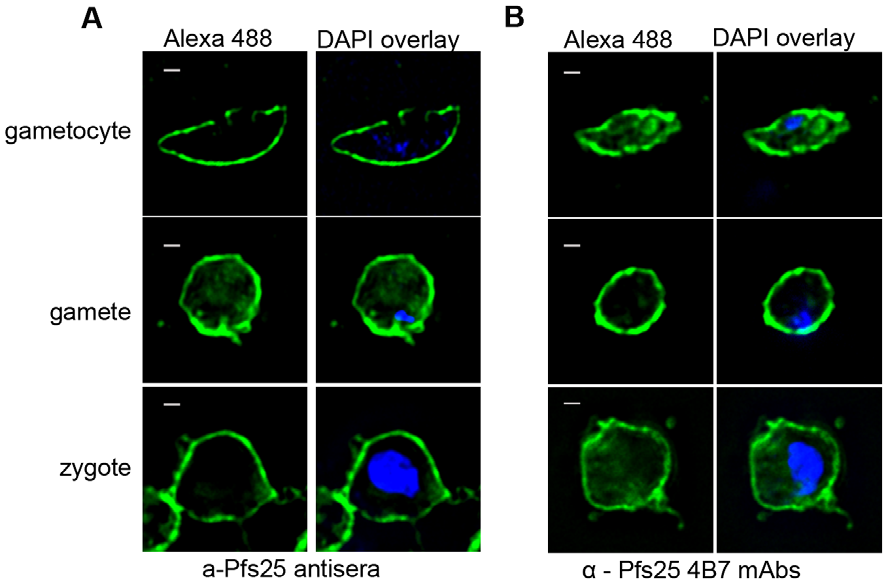
Indirect immunofluorescence using immune sera from mice immunized with algae-produced Pfs25 or Pfs28 on *in-vitro* cultured *P. falciparum* gametocytes, gametes, and zygotes. DNA was stained using DAPI (blue) and antibody binding was visualized using Alexa Fluor 488-conjugated rabbit anti-mouse IgG (green) for (A) a-Pfs25 antisera and (B) anti-Pfs25 4B7 mAbs. Scale bars, 1 µm.

Pfs25 and Pfs28 are outer membrane proteins that are produced in *P. falciparum* sexual stage parasites [39]. We therefore tested the affinity of a-Pfs25 and a-Pfs28 antisera as well as anti-Pfs25-4B7 mAbs and sera from unvaccinated isogenic mice for *in-vitro* cultured parasites. Binding of a-Pfs25 antisera was predominantly confined to the outer membrane(Fig. 6A; additional images Fig. S2). Staining with anti-Pfs25-4B7 was identical to a-Pfs25 antisera (Fig. 6B & Fig. S2), which suggests antibodies to a-Pfs25 also bind Pfs25. Binding of a-Pfs28 antisera was significantly weaker than a-Pfs25 antisera and often not detectable above background levels seen with sera from unvaccinated isogenic mice (Fig. S2). Similar to Western blot analysis of parasite lysates, this suggests that antibody titers to Pfs28 are low or that Pfs28 is not as abundant as Pfs25 in our *in-vitro* cultured parasites.

### Evaluation of transmission blocking activity of a-Pfs25 and a-Pfs28 antisera

**Figure 7:**
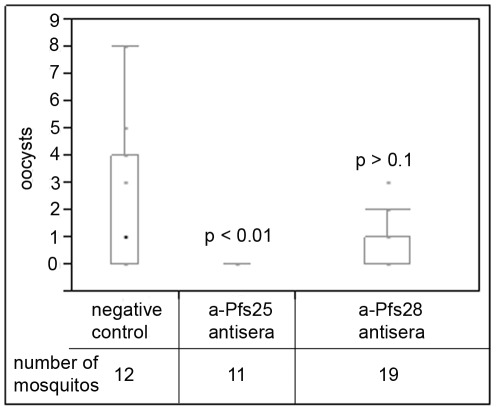
Standard membrane feeding assay with sera from mice immunized with algae-produced Pfs25 or Pfs28. Mosquitos midguts were dissected and analyzed for the presence of oocysts following SMFA. Oocyst numbers are presented as a boxplot. The total number of mosquitos analyzed is listed. Statistics were calculated with a single-tailed Wilcoxon nonparametric comparison.

Transmission blocking activity was measured by the reduction of oocysts in mosquito midguts by a standard membrane feed assay (SMFA). Briefly, *P. falciparum* NF 54 gametocytes and heat inactivated antisera from mice immunized with a-Pfs25, a-Pfs28, or sera from isogenic mice were fed to female *Anopheles stephensi* mosquitoes. Mosquitos were dissected nine days later and analyzed for oocysts (Fig. 7). Oocyst counts were reduced in mosquitos that were fed a-Pfs28 antisera, but transmission blocking activity did not reach significance using a nonparametric comparison test (see materials and methods). Antibodies to a-Pfs25 completely blocked transmission as indicated by the absence of oocysts in all dissected mosquitos. These results are consistent with previous data that suggests that antibodies to Pfs25 more efficiently block malaria transmission than antibodies to Pfs28 [21], [40], and that the algal expressed a-Pfs25 protein elicits an antibody response most suitable for vaccine development.

## Discussion

In this study, we demonstrated that algae are a robust platform for producing malaria subunit vaccines by characterizing Pfs25 and Pfs28, two structurally complex malaria transmission blocking vaccine candidates, made in *C. reinhardtii* chloroplasts. Algae are the only recombinant system to date that has successfully produced unmodified aglycosylated recombinant Pfs25 or Pfs28. The algae-produced recombinant proteins are similar in structure to native Pfs25 and Pfs28 and are recognized by transmission blocking monoclonal antibodies that only bind conformationally correct Pfs antigens. Analysis of free cysteines in a-Pfs25 revealed that disulfide bonds are formed by algal chloroplasts in this protein, but these may not be as extensively formed as in the yeast-produced homologue, Pvs25 from *P. vivax* [35], and this may be an area where the algae system can be improved. However, the disulfide linkages in native Pfs25 are not known, nor have they been characterized in any other recombinant system. The disulfide bonds are likely similar to the native protein because both a-Pfs25 and a-Pfs28 elicit antibodies in mice that recognize native proteins in *P. falciparum* sexual stage lysates. A-Pfs25, but not a-Pfs28, elicited antibodies with significant levels of transmission blocking activity, which is consistent with previous observations [21], [40]. The structural analysis of a-Pfs25 and a-Pfs28 suggests these antigens resemble the native proteins and are of similar conformational quality. The apparent difference in transmission blocking activity between a-Pfs25 and a-Pfs28 is likely due to the delayed appearance of Pfs28 in ookinete development compared to the earlier appearance of Pfs25 in zygote development, rendering any antibodies directed against the later-expressed Pfs28 protein on the parasite superfluous. For these reasons, interest in Pfs28 as a TBV candidate has diminished in recent years compared to Pfs25, which remains the lead TBV candidate.

TBVs and other subunit vaccines must be produced at a cost that is appropriate for low income countries if they are to be implemented. Indeed, financial constraints are already limiting the dissemination of effective meningococcal, pneumococcal, and rotavirus vaccines [41]. Algal biomass could be a low cost source for recombinant protein, especially because recent interest in algal biofuels is driving research in large scale algae cultivation, which is certain to reduce the price of algal biomass production. Recombinant proteins could be separated from lipids used for fuel production, which would drive down the cost of both. Alternatively, fusing mucosal adjuvants to vaccine candidates might allow for oral delivery and eliminate the need for injection and cold chain storage [6], [13], both of which significantly contribute to vaccine cost. Unicellular green algae like *C. reinhardtii* are generally regarded as safe by the U.S. Food and Drug administration because they do not harbor endotoxins, human pathogens, or other known toxic compounds. Therefore, algae may be an ideal platform for producing low-cost subunit vaccines.

## Materials and Methods

### Plasmid construction

The peptide sequences of Pfs25(Ala_22_ – Thr_193_) (Genbank accession: AAD55785) and Pfs28(Val_24_ – Pro_179_) (Genbank accession: AAG27295) were reverse translated with the gene designer algorithm from DNA2.0 (Menlo, CA, USA) using the *C. reinhardtii* complete codon usage table as a reference set (http://www.kazusa.or.jp). The codon bias of the sequence was validated by computing the expected codon adaptation index (eCAI) [26]. The codon optimized algal-pfs25 (*a-pfs25*) and algal-pfs28 (*a-pfs28*) were synthesized by GeneArt®(now Life Technologies Carlsbad, CA, USA) and cloned into the pD1-KanR [11], which contains 5′ and 3′ *psbA* homology to direct homologous recombination to the *psbA* locus and a kanamycin resistance cassette for selection. The resulting plasmids contain a-*pfs25* (pJAG9) or *a-pfs28* (pJAG15) fused to a 3′ TEV protease recognition sequence followed by a FLAG affinity tag. Synthetic *pfs* genes are downstream of the *psbA* promoter and 5′ UTR and upstream of the *psbA* 3′ UTR. Each construct was verified by sequencing (Retrogen, San Diego, CA).

### *C. reinhardtii* transformation

*C. reinhardtii* strain W1.1 [42] was grown in TAP (Tris-acetate-phosphate) medium at 23°C on a rotary shaker to mid log phase and harvested by centrifugation. Approximately 5×10^7^ cells were plated on TAP agar with 100 ug/ml kanamycin and transformed by particle bombardment [43]. Briefly, 1 mg of gold particles (S550d, Seashell technologies, San Diego) coated with 10 ug of plasmid DNA were shot with the PDS-1000/He system (Biorad, Hercules, CA) under vaccum at a distance of 9 cm at 1350 psi. Transformants were propagated on TAP agar with 150 ug/mL kanamycin and screened for the presence of *a-pfs25* or *a-pfs28* using gene specific primers and for homoplasmicity as previously described [44].

### Western blotting, Silver Staining, and affinity purification of a-Pfs25 and a-Pfs28

Initial screens for Pfs protein were performed on homoplasmic strains in 250 ml TAP cultures grown on a rotary shaker. Cultures were grown in low light to mid log phase and then switched to high light (∼5000 lux) conditions. Samples were harvested at various timepoints post-light shift and resuspended in 1 ml lysis buffer per 0.1 g wet algal paste (50 mM Tris pH 8.0, 400 mM NaCl, 0.1% Tween 20, protease inhibitor cocktail (Roche – Mannheim, Germany)). Cells were lysed by sonication and cleared by centrifugation at 30,000×G for 15 minutes. Total and soluble protein samples were prepared in SDS buffer with urea and DTT at a final concentration of 50 mM Tris, 2% SDS, 10% glycerol, 2 M urea, and 100 mM DTT. Samples were heated at 37°C for 10 min then resolved on RUNBLUE precast 12% SDS-PAGE gels (Expedeon – San Diego, CA) and transferred to nitrocellulose membranes. Blots were probed with mouse anti-FLAG primary mAb (Sigma Aldrich – St. Louis, MO) and alkaline phosphatase (AP)-conjugated goat anti-mouse IgG secondary Ab (Sigma-Aldrich) and visualized using nitro-blue tetrazolium (NBT) and 5-bromo-4-chloro-3′-indolyphosphate p-toluidine salt (BCIP) in alkaline phosphatase buffer.

A-Pfs25 and A-Pfs28 were purified from 20 liters of TAP medium grown in photobioreactors. Photobioreactors were constructed from Nalgene carboys (part# – 2251-0050) fitted with Nunc bulkhead adapters (part# – 6149-002) for bubbling air through a 0.1 micron filter. Photobioreactors were inoculated with 250 ml starter cultures at late log phase and grown in the dark until reaching an approximate density of 1×10^6^ cells/mL and then shifted to high light (∼5000 lux). Optimal yields were reached when harvesting 5–8 hrs post-light induction. Cells were harvested using a Lavin L2 continuous flow centrifuge (AML ind. – Hatboro, PA) fed by a peristaltic pump. Total soluble protein was isolated as described above. M2 anti-FLAG resin (Sigma Aldrich) was added to the cleared lysate and rotated end over end at 4°C for 2 hrs. Resin was washed with 20 volumes of lysis buffer twice and once with lysis buffer without Tween 20. Resin was then collected by filtration in a Bio-rad Econo-pac column and the protein was eluted using 100 mM glycine pH 3.5, 400 mM NaCl and neutralized with Tris pH 8.0 to a final concentration of 50 mM. Eluted fractions were resolved by SDS-PAGE and analyzed by Western blot as described above. All fractions containing protein were then combined and buffer exchanged using Vivaspin 6 centrifugal concentrators (GE Healthcare) with a 10 kDa molecular weight cutoff into PBS. The concentration of purified protein was determined using BioRad protein Assay (Biorad). Reduced samples were prepared in SDS buffer with 10% β-mercapotoethanol (BME) and heated to 90°C for 10 min. Non-reduced samples were prepared using 4X Native Buffer (Expedeon). Both reduced and non-reduced samples were resolved on 16% RUNBLUE SDS-PAGE precast gels (Expedeon) for analysis by Western blot. For gel staining, samples were resolved on 4–20% gradient SDS-PAGE precast gels (Biorad) and stained using silver stain plus (Biorad) or Coomassie-blue. Samples for Native-PAGE analysis were prepared with 4X Native Buffer (Expedeon) and resolved by 10% Native-PAGE precast gel (Expedeon) then stained with Coomassie-blue.

Anti-Pfs25 4B7 monoclonal antibody was prepared from mouse hybridoma cell line 4B7.1.1 (ATCC – HB-12575). Cells were grown in hybridoma-SFM media (Gibco, 12045) supplemented with 10% FBS (Gemini) and antibodies were harvested after seven days. Reduced and non-reduced a-Pfs25 and a-Pfs28 were prepared as above. Both were resolved on RUNBLUE 16% SDS-PAGE precast gels (Expedeon) and transferred to a nitrocellulose membrane. The blot was probed using filtered lysate containing anti-Pfs25 4B7 mAbs for a-Pfs25 or anti-Pfs28-2D8. Antibody binding was detected using alkaline phosphatase-conjugated goat anti-mouse secondary (Sigma-Aldrich) and visualized using NBT and BCIP in alkaline phosphatase buffer.

### Circular Dichroism

Far-UC CD spectra were recorded from 190–260 nm with a 0.1 nm data pitch using a Jasco J-815 spectropolarimeter and 1 mm path length. The cuvette chamber temperature was maintained at 20°C by a Jasco PFD-425S/15 temperature unit. Samples were at 21 µM and 27 µM in PBS for Pfs28 and Pfs25, respectively. Spectra were acquired at 1 nm band width, 4 second response time, and a scan speed of 100 nm/min. The results were calculated after subtracting the PBS baseline spectra and reported as mean residue ellipticity. Helical, β-strand, and turn content were predicted using CDSSTR on Dichroweb [36], [37].

### Mass Spectrometry

Pfs25 treated with 0.5 mg/mL of iodoacetamide for 30 min at 37°C to carboxymethylate the cysteine residues that were not in cysteine cross-link bonds. The reaction was quenched with addition of 2 mM DDT followed by buffer exchange using 10 kDa cutoff membrane in 1X TNE buffer (50 mM Tris pH 8.0, 100 mM NaCl, 1 mM EDTA). RapiGest SF reagent (Waters Corp.) was added to the mix to a final concentration of 0.1% and samples were boiled for 5 min. TCEP (Tris (2-carboxyethyl) phosphine) was added to 1 mM (final concentration) and the samples were incubated at 37°C for 30 min. Proteins samples prepared as above were digested with trypsin (trypsin∶protein ratio – 1∶50) overnight at 37°C. RapiGest was degraded and removed by treating the samples with 250 mM HCl at 37°C for 1 h followed by centrifugation at 14000 rpm for 30 min at 4°C. The soluble fraction was then added to a new tube and the peptides were extracted and desalted using Aspire RP30 desalting columns (Thermo Scientific). Trypsin-digested peptides were analyzed by high pressure liquid chromatography (HPLC) coupled with tandem mass spectroscopy (LC-MS/MS) using nano-spray ionization. The nanospray ionization experiments were performed using a TripleTOF 5600 hybrid mass spectrometer (ABSCIEX) interfaced with nano-scale reversed-phase HPLC (Tempo) using a 10 cm-180 micron ID glass capillary packed with 5-µm C18 Zorbax™ beads (Agilent Technologies, Santa Clara, CA). Peptides were eluted from the C18 column into the mass spectrometer using a linear gradient (5–60%) of ACN (Acetonitrile) at a flow rate of 250 µL/min for 1 h. The buffers used to create the ACN gradient were: Buffer A (98% H_2_O, 2% ACN, 0.2% formic acid, and 0.005% TFA) and Buffer B (100% ACN, 0.2% formic acid, and 0.005% TFA). MS/MS data were acquired in a data-dependent manner in which the MS1 data was acquired at m/z of 400 to 1250 Da and the MS/MS data was acquired from m/z of 50 to 2,000 Da. Finally, the collected data were analyzed using MASCOT® (Matrix Sciences) and Protein Pilot 4.0 (ABSCIEX) for peptide identifications.

### Preparation of mouse antisera

Animal experimental protocols were approved by the Institutional Animal Care and Use Committee of the University of California. Balb/c mice were vaccinated by intraperitoneal injection with 25 µg of affinity purified a-Pfs25 or a-Pfs28 emulsified in complete Freund's adjuvant (Sigma-Aldrich). Four boosters were given at two week intervals with 15 µg of affinity purified a-Pfs25 or a-Pfs28 using incomplete Fruend's adjuvant (Sigma-Aldrich). Blood was collected prior to immunization and one week following the final immunization.

### Enzyme-linked immunosorbent assay

The percent of a-Pfs25 and a-Pfs28 of the soluble protein fraction was measured by ELISA. The soluble protein lysates from JAG9 (a-Pfs25) and JAG15 (a-Pfs28) were compared to soluble protein from the untransformed parental strain (W1.1) with a known amount of affinity purified a-Pfs25 or a-Pfs28 as follows. Soluble protein fractions were prepared by sonication followed by centrifugation at 20,000× g. Affinity purified a-Pfs25 or a-Pfs28 was mixed with soluble protein from W1.1 at a 1∶50 ratio, or 2% recombinant protein, and diluted to a final concentration of 0.5 mg/mL in PBS. A standard curve was then prepared using two-fold serial dilutions with a 0.5 mg/ml solution of W1.1 soluble protein. MaxiSorpTM plates (Nunc – Rochester, NY) were coated in triplicate with 50 µg of soluble protein from JAG9, JAG15, or the prepared standard curve and incubated overnight at 4°C on a rocker. Wells were washed three times with PBS with 0.1% Tween (PBS-T) and blocked with 5% milk in PBS-T for 2 hrs at room temperature. A-Pfs25 and a-Pfs28 were detected with M2 anti-FLAG mAbs diluted 1∶4000 in PBS-T and goat anti-mouse horse radish peroxidase (Thermo Scientific – Rockford, IL) at 1∶5000. Antibody binding was detected using the TMB substrate kit (Thermo Scientific) and read with an Infinite M200 pro plate reader (Tecan – Switzerland) at 450 nm. Readings from JAG9 and JAG15 soluble protein were compared to the standard curves prepared with purified protein.

IgG titers in the mouse antisera against a-Pfs25 and a-Pfs28 were measured by ELISA as follows. A-Pfs25 and a-Pfs28 were diluted to 1 µg/ml in PBS and used to coat 96-well MaxiSorp™ plates (Nunc – Rochester, NY) and incubated overnight at 4°C on a rocker. Wells were washed three times with PBS-T and blocked with 5% milk in PBS-T for 2 hours at room temperature. Serum samples were prepared by 1∶5 serial dilutions from 1∶100 to 1∶62500, followed by 1∶2 dilutions from 1∶65000 to 8×10^7^, plated in triplicate, and incubated overnight at 4°C. IgG titers were detected using AP-conjugated goat anti-mouse IgG (Sigma-Aldrich) at 1∶4000 in PBS-T for two hours at room temperature and visualized using p-Nitrophenylphosphate (Enzo Life Sciences – Farmingdale, NY) as the substrate. Absorbances were measured at 450 nm using Infinite M200 pro plate reader (Tecan – Switzerland).

### Western Blotting of parasite lysates

*Plasmodium falciparum* strain NF54 was maintained *in vitro* in continuous cultivation. Gametocytes, macrogametes, zygotes, and ookinetes were cultured and purified as previously described [45]. For antibodies raised to a-Pfs25, 2 µg of reduced and nonreduced parasite lysate was prepared as described above and resolved on RUNBLUE 12% SDS-PAGE precast gels (Expedeon). Resolved proteins were transferred to nitrocellulose and probed with antisera raised to a-Pfs25 at a 1∶100 dilution. Identical blots were prepared and probed with anti-Pfs25 4B7 mAbs or preimmune sera. For antibodies raised to a-Pfs28, 20 µg was prepared in an identical manner and probed with a-Pfs28 antisera at a 1∶100 dilution or preimmune sera. All membranes were visualized using AP-conjugated goat anti-mouse IgG antibodies.

### Indirect Immunofluorescence

Fixed, permeabilized gametocytes, macrogametes, and zygotes were subjected to IFA. Parasites were heat-fixed onto 10-well glass slides (Fisher Scientific). Fixed cells were permeabilized by incubation in PBS containing 3% bovine serum albumin and 0.1% Triton X-100 at room temperature for 15 minutes followed by blocking for 2 hrs at room temperature in 3% BSA in PBS-T. The preparations were incubated with a-Pfs25 or a-Pfs28 antisera (1∶100 dilution) or 4b7 mAbs overnight at 4°C followed by Alexa Fluor 488 goat anti-mouse IgG (1∶500 dilution; Molecular Probes) at room temperature for 1 hr. Parasite nuclei were stained with 2 µg/mL 4′-6-Diamidino-2-phenylindole (DAPI; Molecular Probes) at room temperature for 5 min. Slides were mounted with SlowFade® anti-fade kit (Molecular Probes)and images were acquired using an Applied Precision Spectris microscope and deconvolved using Softworx software (Applied Precision – Issaquah, Washington). Images were adjusted for contrast in Softworx and exported as tiffs and assembled with Adobe Photoshop.

### Standard Membrane Feeding Assay

Transmission blocking activity was assessed by standard membrane feeding [46]. *Anopheles stephensi* STE2 was maintained at 27°C and 80% relative humidity on a 12 hr day/night light cycle. Larvae were fed a diet of powdered fish food (Tetramin) mixed with yeast. Adults were provided a 10% sugar solution. Four to six day-old female *A. stephensi* mosquitoes were fed with *P. falciparum* NF 54 gametocytes in the presence of heat inactivated control or immune sera or mAbs using a membrane feeding apparatus. Heat inactivation was performed at 56°C for 45 min. After 15 min of feeding, un-engorged mosquitoes were removed and engorged mosquitoes were maintained in the insectary under standard conditions [47]. Midguts were dissected 9 days after the infectious bloodmeal, stained with 0.1% mercurochrome and the number of oocysts in each preparation counted. Uninfected bloodmeals were provided to wild-type control mosquitoes following the membrane feeding. Infected female mosquitoes were dissected for oocyst counts nine days after infection. Statistics were calculated in JMP ver. 9.0.2 (SAS – Cary, NC) using a single tailed Wilcoxon nonparametric comparison.

## Supporting Information

Figure S1 **Silver stain analysis of algae-produced Pfs25 and Pfs28.** Increasing amounts of affinity purified a-Pfs25 and a-Pfs28 (100, 200, and 400 ng) and 200 ng of BSA were resolved on 16% SDS-PAGE and total protein was detected using silver stain.
(TIF)

Figure S2 **Indirect immunofluorescence using immune sera from mice injected with algae-produced Pfs25 or Pfs28 on** ***in-vitro*** **cultured** ***P. falciparum*** **gametocytes, gametes, and zygotes.** DNA was stained using DAPI (blue) and antibody binding was visualized using Alexa Fluor 488-conjugated rabbit anti-mouse IgG (green) for a-Pfs25 antisera, a-Pfs28 antisera, anti-Pfs25 4B7 mAbs, and sera from isogenic unvaccinated mice.
(TIF)
