# Supplementary figures and images for: Algae-Produced Pfs25 Elicits Antibodies That Inhibit Malaria Transmission
Source: PLoS One. 2012 May 16;7(5):e37179. doi: 10.1371/journal.pone.0037179 (PMC3353897; doi:10.1371/journal.pone.0037179)

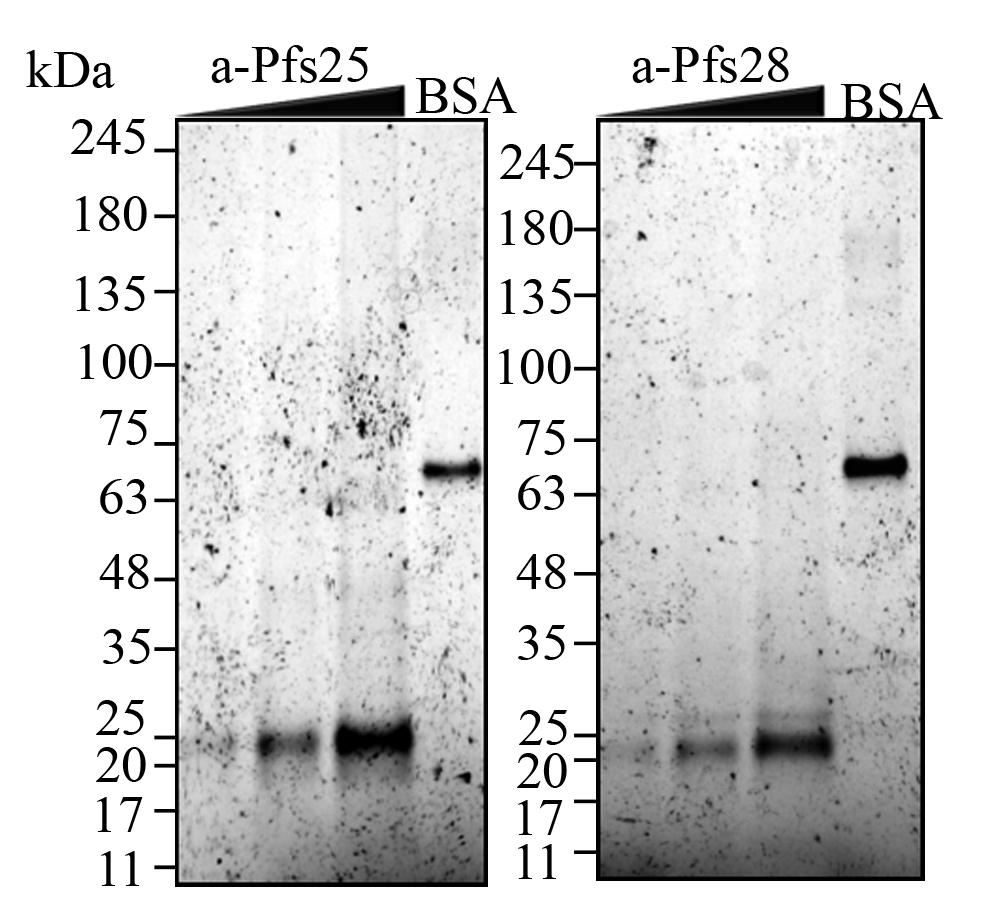

Supplement: Figure S1 — Silver stain analysis of algae-produced Pfs25 and Pfs28. Increasing amounts of affinity purified a-Pfs25 and a-Pfs28 (100, 200, and 400 ng) and 200 ng of BSA were resolved on 16% SDS-PAGE and total protein was detected using silver stain. (TIF) [file pone.0037179.s001.tif]

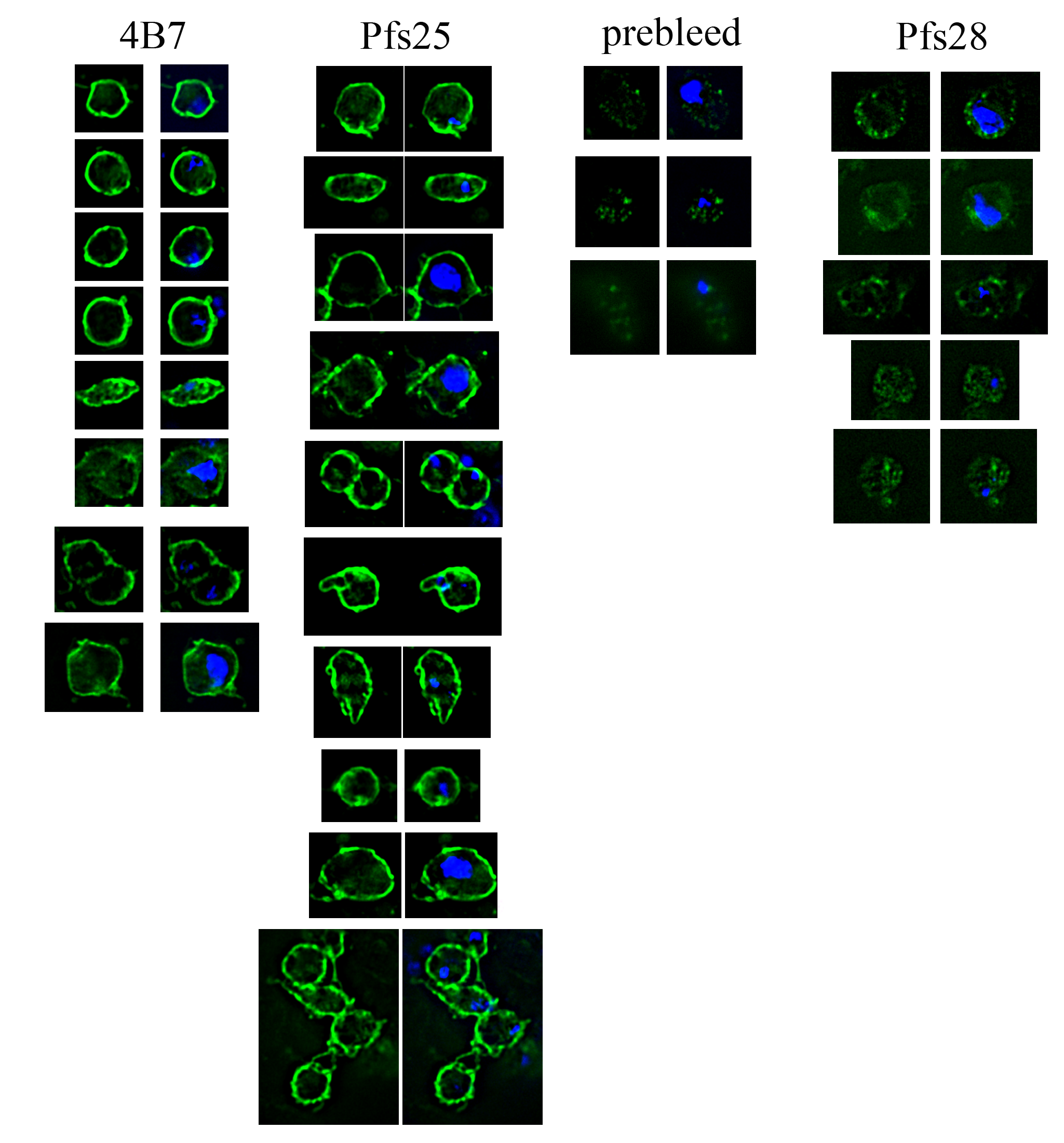

Supplement: Figure S2 — Indirect immunofluorescence using immune sera from mice injected with algae-produced Pfs25 or Pfs28 on in-vitro cultured P. falciparum gametocytes, gametes, and zygotes. DNA was stained using DAPI (blue) and antibody binding was visualized using Alexa Fluor 488-conjugated rabbit anti-mouse IgG (green) for a-Pfs25 antisera, a-Pfs28 antisera, anti-Pfs25 4B7 mAbs, and sera from isogenic unvaccinated mice. (TIF) [file pone.0037179.s002.tif]
